# Acute Myopericarditis Secondary to Chagas Disease

**DOI:** 10.7759/cureus.46301

**Published:** 2023-10-01

**Authors:** Ivan A Elizalde Uribe, Maria F Osorno Gonzalez de Leon, Karla V Barrios Perez, Diana C Valle Robles, Bruno A Lopez-Luis, Elias N Andrade Cuellar, Sandra I Dominguez Valdez, Juan E Muñoz Arellano, Deyanira Alvarez Camargo, Gabriela Melendez Ramirez

**Affiliations:** 1 Internal Medicine, Centro Medico Nacional "20 de Noviembre", Mexico City, MEX; 2 Cardiology, Centro Medico Nacional "20 de Noviembre", Mexico City, MEX; 3 Radiotherapy, Centro Medico Nacional "20 de Noviembre", Mexico City, MEX; 4 Infectious Disease, Instituto Nacional de Ciencias Médicas y Nutrición Salvador Zubirán, Mexico, Mexico City, MEX

**Keywords:** magnetic resonance (mr), successfully treated myopericarditis, acute myopericarditis, chagas diagnosis, chagas cardiomyopathy

## Abstract

American trypanosomiasis or Chagas disease is predominantly a vector-borne multisystemic infection caused by *Trypanosoma cruzi*, a protozoan parasite transmitted by triatomine bugs in endemic areas^ ^such as Mexico and Central and South America. Acute *T. cruzi *infection is mostly asymptomatic, nonetheless, in up to one-third of the patients, a mild form of the disease can be present, with nonspecific manifestations like fever, lymphadenopathy, hepatosplenomegaly, inflammation at the inoculation site (inoculation chancre) and unilateral palpebral edema (Romaña sign). Severe acute disease occurs in less than 1% of patients and includes myopericarditis and meningoencephalitis. If untreated, the acute phase can cause chronicity with cardiac and gastrointestinal involvement. We report the case of a female with occupational exposure to this parasite, who presented with acute myopericarditis, a rare form of presentation of this disease.

## Introduction

American trypanosomiasis or Chagas disease is a pathology caused by the protozoan *Trypanosoma (T.)* cruzi and is an entity with variable clinical presentation and multisystem involvement that can involve the cardiovascular system [1,2]. The incubation period for *T. cruzi* varies from one to two weeks after vector-borne transmission. Two-thirds of infected patients remain asymptomatic during their life [2].

The acute phase of Chagas disease has been reported to last from eight to 12 weeks and is rarely diagnosed because it is mostly asymptomatic, but in some cases, it can present with nonspecific symptoms such as fever, malaise, and splenomegaly. It has been reported that approximately 30% of infected patients can develop a chronic phase of this disease, with damage to specific organs [1].

The main transmission mechanism of Chagas disease in endemic areas is mediated by triatomines [1]. There are other infection mechanisms that are important, especially in non-endemic areas, including blood transfusion, organ transplantation, oral ingestion, vertical transmission from mother to child, or the use of shared intravenous needles [1]. Laboratory accidents are a rare form of transmission [3]; however, this was considered to be the contagion mechanism in our patients.

## Case presentation

A 37-year-old female, with no previous medical history, was admitted to the Internal Medicine department with a three-week history of malaise, myalgia, bilateral joint pain in the ankles and knees, and intermittent fever up to 39º C (102.2ºF). The patient self-medicated with oral acetaminophen 500 mg every eight hours and intramuscular dexamethasone 8 mg daily for four days, with partial improvement of the symptoms. The patient referred to working as a researcher in a Tropical Diseases laboratory with animal models and handling rabbits and mice daily. She does not live in an endemic area of this disease.

One week before the admission, she complained of sore throat and dry cough associated with exertional dyspnea, with deterioration of functional class from New York Heart Association (NYHA) I to NYHA II, palpitations, tachycardia of up to 130 beats per minute, and edema in the lower extremities. She also noted a slightly itchy diffuse rash that vanished after three days. She denied headaches, visual disturbances, dysphagia, chest pain, or any other symptoms.

Her blood pressure was 105/68 mmHg, pulse 114 beats per minute, respiratory rate of 18 breaths per minute, temperature of 37.9°C (100.2°F), and oxygen saturation of 95% on room air.

Physical examination revealed rhythmic heart sounds, with an S1 and S2 present, and pericardial friction rub but the rest of the precordial examination was otherwise normal, with no murmurs or gallops. There was no increased work of breathing, lungs were clear to auscultation bilaterally, and no wheezes or crackles were found. Discrete hepatomegaly was found with a non-palpable spleen. Signs of fluid overload were evidenced by jugular venous distention, hepatojugular reflux, and bilateral leg swelling, particularly in the pretibial region and ankles. No lymph node enlargement was detected. No neurological or joint abnormalities. There was no evidence of scars on the hands. The rest of the exam was unremarkable.

On laboratory results, liver enzymes were elevated and cardiac biomarkers values were above the reference range, all laboratory values are reported in Table 1.

**Table 1:** Laboratory values NT-proBNP: N-terminal pro-B-type natriuretic peptide

Parameter	Result	Reference Value
Aspartate aminotransferase	131 U/L	0 – 34 U/L
Alanine aminotransferase	128 U/L	10 – 49 U/L
Albumin	2.3 g/dL	3.2 – 4.8 g/dL
Lactate dehydrogenase	466 UI/L	120 – 246 UI/L
D-dimer	2.29 mg/L	0.05 – 0.26 mg/L
C-reactive protein	149 mg/L	0 – 3 mg/L
Erythrocyte sedimentation rate	41 mm/hr	0 – 10 mm/hr
Lactate	3.4 mmol/L	0.5 – 2 mmol/L
Troponin I	0.47 ng/ml	< 0.08 ng/mL
Creatine phosphokinase	140 UI/L	34 – 145 UI/L
Creatine phosphokinase-MB	25 UI/L	0 – 14 UI/L
Myoglobin	85 ug/L	10 - 46 ug/L
NT-proBNP	865 pg/mL	< 125 pg/mL

An echocardiogram was performed that reported the ventricle without motility alterations at rest, not dilated, with a systolic function of 70% (global longitudinal strain (GLS) - 12.4%), normal diastolic function, and the right ventricle and both atria normal, without pericardial effusion.

**Figure 1:**
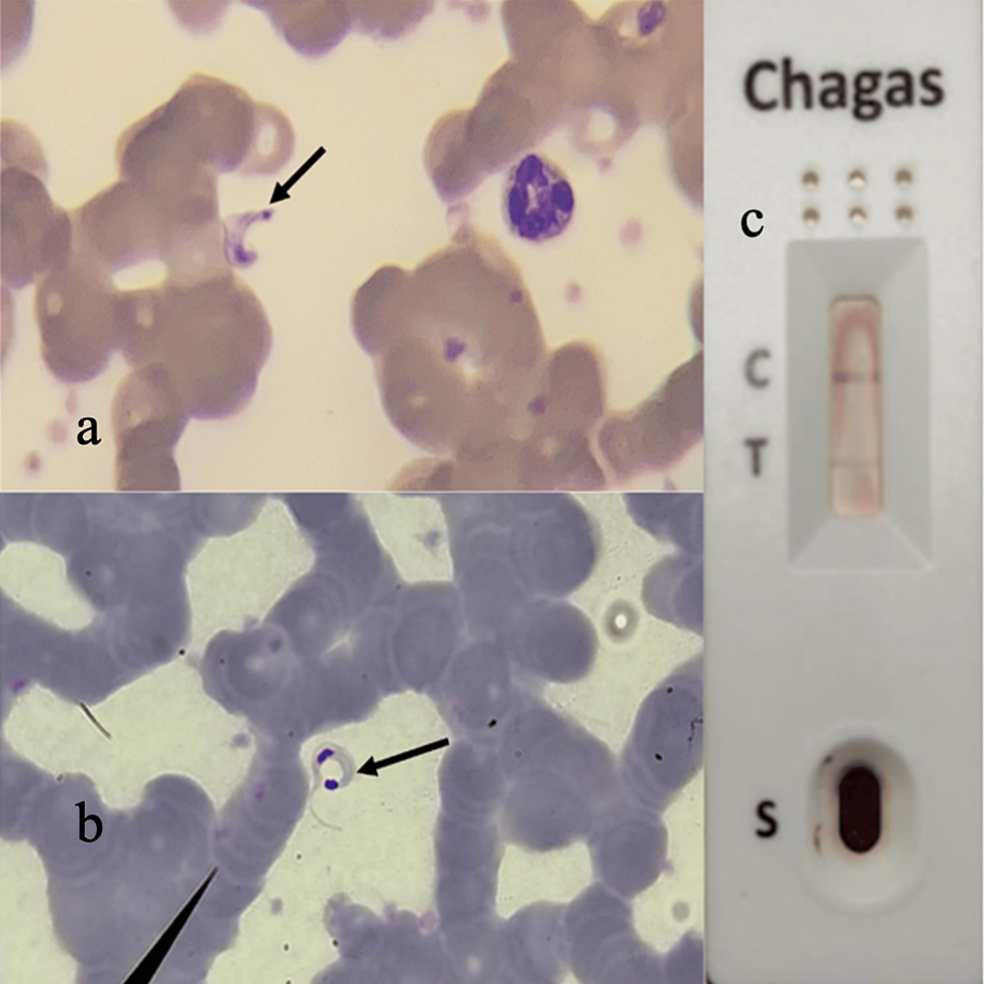
Complementary Studies a) Peripheral blood smear in the presence of trypomastigote; b) Thick peripheral blood smear stained with Giemsa showing trypomastigote; C) Positive IgM serology against *T. cruzi* in peripheral blood

Due to occupational exposure to parasites, a rapid immunofluorescence immunoglobulin G (IgG) test for *Trypanosoma cruzi *was carried out with a positive result (Figure 1). Diagnosis of acute Chagas disease was confirmed by the presence of trypomastigotes on a Giemsa-stained thick blood smear and a qualitative polymerase chain reaction (PCR) assay.

**Figure 2:**
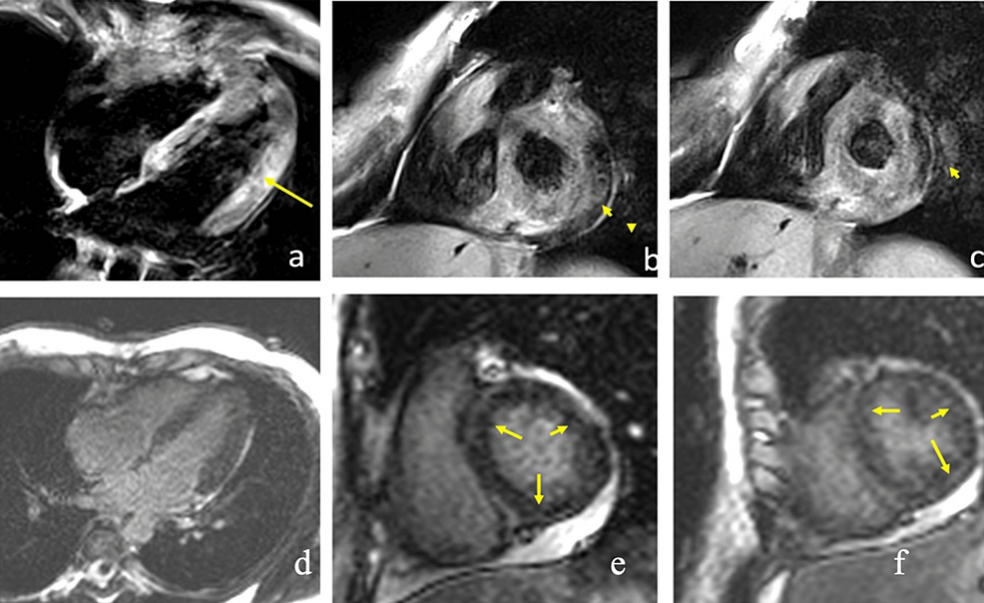
Cardiac MRI Four-chamber T2-weighted sequence (a), basal third short axis (b), and half (c). The arrow points to the lateral hyperintensity of the middle third and the arrowhead to the pericardium adjacent to the inferior and lateral wall. In d-f, the inversion-recovery sequence in the same planes as a-c is seen; the arrow indicates the global intramyocardial late enhancement of the left ventricle, predominantly basal.

Findings on cardiac magnetic resonance imaging (cardiac MRI) met the 2018 Lake Louise criteria for acute myocarditis. The study exhibited inferior pericardial effusion of 12 mm, and late gadolinium enhancement (LGE) on the parietal pericardium and on the septal, inferior, and lateral myocardium (Figure 2). The T2-weighted sequence showed pericardial, basal inferoseptal, and anterolateral myocardial enhancement. Left ventricular ejection fraction (LVEF) was 63%.

Pharmacological treatment with acetylsalicylic acid 1 g every eight hours and colchicine 0.5 mg every 24 hours was started and the patient was treated with oral furosemide for five days with an effective reduction of leg edema. After six days of hospitalization, she was discharged and started on oral nifurtimox (8 mg/kg/day in two divided doses) for eight weeks. By the second week, she reported a general improvement in symptoms but complained of mild abdominal discomfort associated with treatment. Tolerance and adherence were otherwise adequate. Liver and cardiac biomarkers were within the normal ranges by the third week, and she remained afebrile until the end of the treatment.

When treatment was completed, a new cardiac MRI was performed. Pericardial effusion and enhancement on the T2-weighted sequence were no longer observed, although focal inferior myocardial LGE remained. LVEF was 61%. Ambulatory ECG monitoring was normal. Follow-up PCR and thick blood smears were negative at weeks 4 and 8.

## Discussion

Chagas cardiomyopathy encompasses all cases of Chagas disease with cardiac involvement, according to the American Heart Association. This is defined by the presence of at least one typical electrocardiographic abnormality in those patients who have positive serological tests against *T. cruzi* [2].

In the acute phase, mild cardiac abnormalities, such as disproportionate tachycardia, are often noted [2]. ECG during mild acute illness shows sinus tachycardia and PR/QT prolongation, low-voltage QRS complexes, and repolarization abnormalities [4]. When there are more advanced electrocardiographic findings, such as right bundle branch block, atrial fibrillation, or ventricular arrhythmias, they are indicators of a worse prognosis [2,5].

Acute infection usually resolves spontaneously [1]. However, 20% to 40% develop cardiomyopathy 20 to 30 years later at a rate of 3-4% per year [1,4]. Unfortunately, in many cases, the diagnosis is made at a late stage, due to the non-specific nature of the disease and the limited availability of the studies required to diagnose it [3,5,6].

In the acute phase, the diagnosis of cardiovascular disease is achieved in only 1% to 2% of patients [5]. Trypanocidal therapy with benznidazole or nifurtimox results in a reduction in detectable parasitemia, with up to 85% of patients receiving parasitological cure [6].

The elevation of BNP or NT-proBNP is observed in some cases before advanced cardiac disease, these elevations can be subtle, and inconsistent and have not been shown to be clinically prognostic [2]. However, increased BNP is a good marker of the presence of LV systolic and diastolic dysfunction in Chagas cardiomyopathy [2,6].

Magnetic resonance imaging has stood out as support in the diagnosis of cardiac disease due to its ability to identify myocardial fibrosis, edema, and hyperemia, allowing the identification of a significant percentage with silent presentation as indeterminate phases due to late gadolinium enhancement (LGE), as well as the acute condition in integration with the serological tests [2,4].

It is characteristic that this cardiomyopathy has a high formation of collagen bands, creating an intense structural modification [4]. Due to this, magnetic resonance imaging has been considered as a study that can non-invasively demonstrate this structural disorder associated with high collagen formation [4].

The importance of magnetic resonance imaging on the prognosis of arrhythmias and sudden death in the acute phase has recently been demonstrated by analyzing the presence and pattern of LGE in these patients [4].

## Conclusions

Given the low rate of diagnoses that are made in the acute phase of this disease, we believe it is important to report the imaging characteristics found by magnetic resonance imaging in our case, which can serve as a prognostic factor for the development of long-term complications in the patient.

Treatment during the acute phase offers a better prognosis, achieving cure rates between 65% and 80%, as occurred with this patient in whom a good clinical response with parasitologic cure was observed.
